# The Safety and Efficacy of Antifibrinolytic Therapy in Neonatal Cardiac Surgery

**DOI:** 10.1371/journal.pone.0126514

**Published:** 2015-05-08

**Authors:** Chih-Yuan Lin, Jeffery H. Shuhaiber, Hugo Loyola, Hua Liu, Pedro del Nido, James A. DiNardo, Frank A. Pigula

**Affiliations:** 1 Department of Cardiac Surgery, Children’s Hospital Boston, Harvard Medical School, Boston, Massachusetts, United States of America; 2 Department of Anaesthesia, Children’s Hospital Boston, Harvard Medical School, Boston, Massachusetts, United States of America; 3 Division of Cardiovascular Surgery, Department of Surgery, Tri-Service General Hospital, National Defense Medical Center, Taipei, Taiwan; University of Tübingen, GERMANY

## Abstract

**Background:**

Neonates undergoing open-heart surgery are particularly at risk of postoperative bleeding requiring blood transfusion. Aprotinin has attained high efficacy in reducing the requirement for a blood transfusion following a cardiopulmonary bypass, but is seldom studied in the neonatal age group. The aim of this study was to compare the efficacy and adverse effects of aprotinin and tranexamic acid in neonates undergoing open-heart surgery at a single centre.

**Methods:**

Between October 2003 and March 2008, perioperative data of 552 consecutive neonatal patients undergoing open-heart surgery in Children’s Hospital Boston were reviewed. Among them, 177 did not receive antifibrinolytic therapy (Group A); 100 were treated with tranexamic acid only (Group B); and 275 patients received aprotinin with or without tranexamic acid (Group C). Except for antifibrinolytic therapy, the anaesthesiological and surgical protocols remained identical. Postoperative complications and in-hospital mortality were the primary study endpoints.

**Results:**

Body weight and Risk Adjustment for Congenital Heart Surgery (RACHS-1) scores were statistically comparable among the three groups. No statistically significant differences were observed between the duration of hospitalization, chest tube drainage, reexploration for bleeding, and kidney function impairment. In Group C, less blood was transfused within 24 hours than in GroupB. Operative mortality was similar among the three groups.

**Conclusion:**

No further risk and kidney injury were observed in the use of aprotinin in neonatal cardiac surgery, aprotinin demonstrated a reduced requirement for blood transfusion compared with tranexamic acid. Our data provide reasonable evidence that aprotinin and tranexamic acid are safe and efficacious as antifibrinolytic modalities in neonatal patients undergoing cardiac surgery.

## Introduction

Cardiopulmonary bypass (CPB) and cardiovascular surgery activate coagulation, inflammation, and fibrinolysis, which often exert potentially deleterious effects on patient outcome, particularly if CPB is prolonged [1, 2]. The use of CPB to repair congenital cardiac defects among neonates often subjects these young patients to wide ranges of physiologic derangement because of immature tissue and organ function in the first month of life. Neonates often experience more pronounced deleterious effects than those seen in large paediatric or adult patients, partially because of the substantial disparity in CPB circuit size [3]. Although haemodilution can produce impaired haemostasis related to both qualitative and quantitative abnormalities in coagulation factors, leading to fibrinolysis [2–6], antifibrinolytics can be helpful in reducing loss and transfusion requirements [7, 8].

Aprotinin (Trasylol; Bayer Pharmaceuticals Corporation, West Haven, CT, USA) is a nonspecific serine protease inhibitor that was first used as an antiinflammatory agent in the treatment of acute pancreatitis in the 1960s. The beneficial effects of aprotinin in open-heart surgery reportedly work by inhibiting kallikrein and plasmin, with decreased haemostatic activation, inhibition of fibrinolysis, and preservation of platelet function [9]. In a randomized controlled trial published in 1987, aprotinin was found to reduce blood loss and the need of transfusion in re-do open heart surgery [10]. In the following days, several randomized, prospective, placebo-controlled, carefully performed trials on aprotinin use have indicated a reduced blood transfusion requirement in adult cardiac surgery [11]. By 1993, the Food and Durg Administration (FDA) approved the use of aprotinin in coronary artery bypass grafting for reducing blood loss [12]. Additionally, aprotinin has also reportedly decreased the inflammatory response to CPB, verified by reduced levels of proinflammatory cytokines in aprotinin-treated patients [13, 14]. In 2008, the results of the Blood Conservation Using Antifibrinolytics in a Randomized Trial (BART) were published [15]. BART was a multicentre, blinded, randomized trial of 2331 high-risk cardiac surgery patients comparing aprotinin with a pair of lysine analog drugs (tranexamic acid and epsilon-aminocaproic acid) owing to concerns of an increased risk of deaths related to aprotinin. From the BART and other observation studies [16–18], aprotinin has been associated with greater risk to benefit ratio among adult populations and the safety concern of aprotinin increased [19]. Consequently, aprotonin was withdrawn and has been clinically unavailable in the United States since 2007. However, because of the biological and procedural differences between surgery for congenital and acquired cardiac disease, the paediatric experience remains relevant. Aprotinin reportedly decreased the use of blood products and operative time in congenital heart surgery [20]. Additional benefits in congenital heart surgery include improved pulmonary function, particularly in patients undergoing surgical palliation for single ventricle anatomy [21]. However, data regarding the safety and efficacy of aprotinin in neonatal patients are limited.

Tranexamic acid, a lysine analogue, is widely used to reduce blood loss; however, the blood-sparing effect is often considered to be inferior to that of aprotinin [22]. Additionally, the safety of aprotinin has been more extensively studied than that of tranexamic acid. A previous study on paediatric cardiac surgery demonstrated increased susceptibility to seizure after tranexamic acid treatment [23].

The purpose of this study was to determine the safety and efficacy of aprotinin and tranexamic acid in neonatal patients undergoing CPB.

## Patients and Methods

The Institutional Review Board of Children’s Hospital Boston, Boston, Massachusetts, United States approved this retrospective, nonrandomised cohort study, and the approval included a waiver of informed consent. Data were collected from a retrospective review of prospectively collected data. From October 2003 to March 2008, 552 neonatal patients undergoing 565 cardiac surgical procedures were enrolled in this study. Neonates were divided into three groups: Group A: no antifibrinolytic group (n = 177); Group B: tranexamic acid group (n = 100); and Group C: aprotinin or both aprotinin and tranexamic acid (n = 275). Demographic data were recorded, including gender, weight and age at surgery, and prematurity. Intraoperative information included deep hypothermic circulatory arrest, aortic cross-clamp time, CPB time, use of Risk Adjustment for Congenital Heart Surgery (RACHS-1) scores to assess the complexity of the procedures performed, and duration of hospitalization in days (ICU and ward). Renal function was assessed by measuring pre- and postoperative serum creatinine (Cr). Requirements for extracorporeal membrane oxygenation (ECMO), reoperation for bleeding, blood transfusion within 24 hours and in-hospital death were recorded and analysed.

The protocol consisted of administering a bolus of 100 mg/kg of tranexamic acid to the patient after induction of anaesthesia, followed by an infusion of 10 mg/kg/h until the end of the operation, and 100 mg/kg was added to prime the CPB equipment. In the aprotinin group, a bolus of 30,000 KIU/kg of aprotinin was administered to patients after the induction of anaesthesia, followed by an infusion of 10,000 KIU/kg/h until the end of the operation, and 30,000 KIU/kg was also added to the CPB prime.

In all other aspects, the surgical and anaesthesiological protocols remained unchanged. The requirement of a transfusion was considered when the haemoglobin level was < 14 g/dL in cyanotic patients, and < 10 g/dL in noncyanotic patients, or if a patient exhibited clinical signs indicating the requirement for a higher oxygen carrying capacity. Reexploration for mediastinal bleeding was based on clinical signs, including excessive chest-tube output. We did not have a set criteria for exploration regarding chest-tube blood output.

Chart reviews consisted of preoperative, intraoperative and postoperative data collection. Preoperative data included patient demographics: age, gender, weight, prematurity (defined as < 36 wk gestation), and presence of a major noncardiac structural anomaly. Cardiac diagnosis and surgical procedure were recorded. All neonates were assigned a RACHS-1 score [24]. Neonates with a combination of cardiac surgical procedures were assigned the RACHS-1 score of the highest risk procedure and the combination of procedures was treated as an independent variable. Preoperative baseline creatinine (Cr) was also recorded. Intraoperative data included the use of aprotinin or tranexamic acid, CPB time, aortic cross-clamp time, and deep hypothermic circulatory arrest time. Postoperative data were used to assess specific outcomes. Postoperative Cr levels were recorded at 24 hours. Biochemical acute kidney failure was defined as an increase in serum creatinine levels to double or more than the preoperative level. We also recorded the time to tracheal extubation and the duration of the intensive care unit stay, the duration of chest-tube drainage, the use of ECMO and mortality before hospital discharge.

The data included in our manuscript represents an entirely clinical data base obtained from the department of surgery databases, without the reliance of administrative databases, and included discrete information not available in previous publications.

## Data analysis

The three groups of patients were compared using the preoperative, intraoperative, postoperative complications results. Collected data were summarized descriptively and compared between each two of the three groups. The Shapiro-Wilk test was used to assess the normality distribution assumption. Data were described using means and standard deviation, and comparisons were performed using an analysis of variance and a posthoc test if appropriate. Nonnormally distributed data were compared using the Kruskal-Wallis equality-of-populations rank test and a posthoc test for multiple comparisons, as described in Siegel and Castellan [25]. Categorical data were analysed using Fisher’s exact test. Outcome data was presented using Kaplan-Meier survival curves with a comparison between types of antifibrinolytic therapy by using a log-rank test. A multivariable logistic regression model was used to evaluate the association between antifibrinolytic therapy and death adjusted for a previously described set of covariates. The goodness of fit was evaluated using the Hosmer-Lemeshow chi-square. All statistical analysis was performed using the STATA/IC 10.1 (STATA Corp, College Station, TX, USA) software and statistical significance was assessed at the .05 level.

## Results

**Fig 1:**
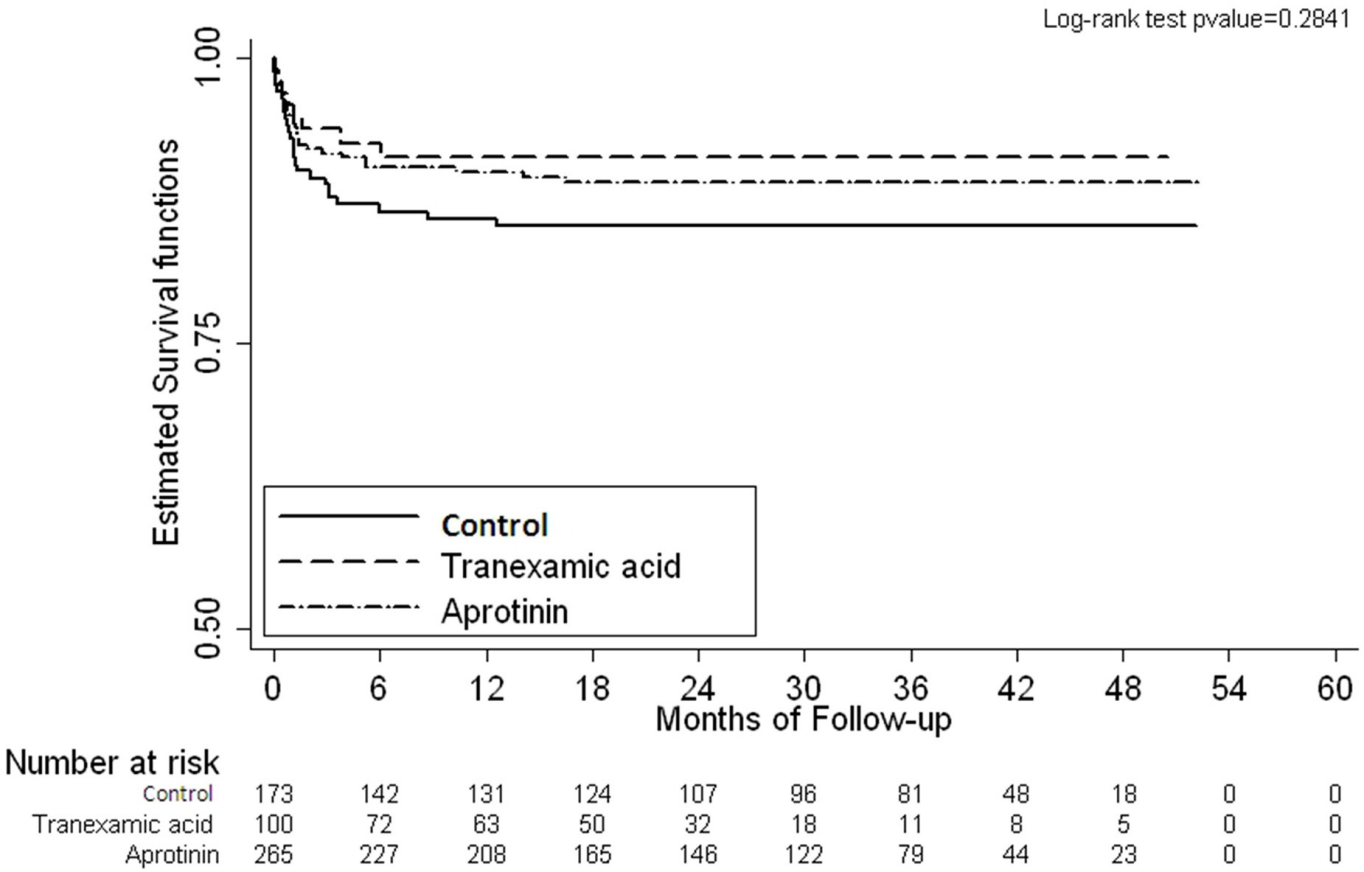
Kaplan-Mier survival curve.

Male patients predominated in all three groups. The demographic and intraoperative data are listed in Table 1. Weight and the percentage of prematurity were comparable across the three groups and no significant difference was observed in the RACHS scores. The K-W test indicated that the median deep hypothermic circulatory arrest (DHCA) time in Group C was significantly lower than that in Groups A and B (P = .00010). The median CPB time differed significantly among the groups (P = .00275): between the control and tranexamic acid groups (P = .005419), and the control and aprotinin groups (P = .00063). The total blood transfusion within 24 hours after surgery (excluding reoperation for bleeding) varied among all three groups (P = .0011) (Table 2). Chi-square analysis revealed no statistical differences in the incidence of acute kidney injury among the three groups (P = .655), and the odds ratio for death was similar (Table 3). The estimated survival functions for death and being reoperation-free between the antifibrinolytic therapy groups were also similar (P = .2841 and .2897) (Fig 1).

**Table 1:** Preoperative and intraoperative characteristics.

Characteristic	Group ANo antifibrinolytic(n = 184)	Group B Tranexamic acid only(n = 104)	Group C Aprotinin and both aprotinin and tranexamic acid(n = 276)	p-Value
Patients, n	177	100	275	
Male gender, n(%)	105 (59.3)	56 (56.0)	155 (58.4)	0.87
Age at surgery median (IQR) days	6 (4–12)	6 (4–12)	5 (4–8)	0.0036
Weight, mean (SD) Kg	3.06 (0.61)	3.26 (0.56)	3.10 (0.66)	0.0520
Prematurity, n	3	0	10	0.138
RACHS-1 score, median (IQR)	3 (3–6)	3 (3–4)	4 (3–6)	0.2850
Deep hypothermic circulatory arrest, median (IQR) min	32 (13–48)	31.5 (12.5–49)	17 (9–27)	0.0001
CPB time, median (IQR) min	128 (100–158)	142 (116–166)	138 (120–163)	0.0027
Aortic cross-clamp time, median (IQR) min	65 (48–94)	78 (54–99)	71 (57–86)	0.0971

CPB: Cardiopulmonary bypass; IQR: Interquartile range

**Table 2:** Postoperative characteristics and morbidity/mortality.

Characteristic	Group ANo antifibrinolytic(n = 184)	Group B Tranexamic acid only(n = 104)	Group C Aprotinin and both aprotinin and tranexamic acid(n = 276)	p-Value
Total blood transfusion within 24 h, mean (STD) ml	39.8±47.0	47.7±51.0	32.0±40.0	0.011
ICU LOS, median (IQR) days	11 (7–19)	13 (9–20)	11 (9–18)	0.6395
Hospital LOS (days), median (range)	16 (12–28)	19(12–34)	16(16–26)	0.2575
Chest tube drainage (days), median (range)	4 (1–63)	4(1–63)	4(1–44)	0.5324
Acute kidney injury, n (%)	16 (8.7%)	10 (9.6%)	31 (11.2%)	0.655
Reopen chest, n (%)	9 (4.9%)	7 (6.7%)	10 (3.6%)	0.4270
ECMO support, n (%)	2 (1.1%)	0 (0%)	7 (2.5)	0.1690
Hospital mortality, n (%)	29 (15.8%)	8 (7.7%)	28 (10.1%)	0.072

**Table 3:** Adjusted effect of antifibrinolytic therapy on odds ratio for death.

Risk factor	OR	P value	[95%CI for OR]
None	1.00*		
Transexamic Ac	0.27	0.092	0.06–1.23
Aprotinin	0.31	0.357	0.02–3.62
Gender(female ref)	1.27	0.720	0.33–4.79
Age at surgery	1.05	0.240	0.96–1.16
Days in Hospital	0.77	0.001	0.67–0.89
Days in CICU	1.35	<0.001	1.16–1.57
Cross-clamp Ao	0.96	0.039	0.93–0.99
CPB temp	1.21	0.012	1.04–1.41
CPB time	1.02	0.020	1.00–1.05
RACHS-1 level 1–2	1.00*		
RACHS-1 level 3	1.28	0.816	0.15–10.4
RACHS-1 level 4	0.73	0.831	0.04–12.6
RACHS-1 level 6	7.31	0.084	0.76–69.9
Creatinine pre-post	3.74	0.385	0.19–73.9
Transfusion < 24h	0.98	0.200	0.97–1.00

*Reference group of the following OR.

Hosmer-Lemeshow chi^2^ (8) = 3.73Prob > chi^2^ = 0.8805

## Discussion

Congenital heart disease (CHD) has long been associated with coagulation abnormalities [26], including platelet abnormalities [27] and fibrinolysis [28]. To overcome these adverse effects, attempts have been made to modify these effects with both lysine analogue, antifibrinolytics, and aprotinin. Aprotinin is a broad-spectrum serine protease inhibitor that protects platelets by preventing their activation on CPB [29]. Previous reports focusing on aprotinin in patients with various congenital heart diseases who underwent cardiac operation have consistently revealed a decrease in postoperative blood loss [30]. However, there is no definite conclusion regarding the efficacy of antifibrinolytic therapy in neonatal patients undergoing cardiac operation. Because analyses of the effects on paediatric cardiac operations have been hampered by various confounding factors, undefined transfusion triggers, various dosing protocols and institutional preference, information regarding the efficacy of aprotinin in neonates remains limited and occasionally conflicting [31, 32]. In our study, using specified transfusion triggers, blood transfusion within 24 hours was significantly less common among those who received aprotinin (32.0 ± 40.0 mL) compared with the other two groups (P = .011) (39.8 ± 47.0 mL in Group A and 47.7 ± 51.0 mL in Group B).

Numerous studies have reported findings demonstrating increased mortality and renal failure in cardiac surgery patients [15,19]. Aprotinin was suspended at the FDAs request and removed from the market in 2007. Some publications suggested that the withdrawal of aprotinin has been detrimental to patients undergoing cardiac surgery due to increased adverse outcomes and use of blood products [33–35], but others found a relatively minimal influence in clinical practice [36]. However, no randomized multicentre study has demonstrated any robust adverse events in a paediatric group, particularly among neonates undergoing cardiac surgery [37–40]. Because of the concern that children undergoing congenital heart operations are more prone to bleeding than adults are [41], tranexamic acid is considered the second most suitable alternative to antifibrinolytic therapy. Schindler et al. reported that aprotinin can be replaced with tranexamic acid because of the equal blood sparing effects, but they also indicated that the evidence accumulated at present remains insufficient to clearly estimate the benefits and risks associated with the use of tranexamic acid in congenital heart surgery [42].

In addition to its antifibrinolytic property, aprotinin might exert an attenuating effect on the inflammatory response to CPB. This effect can be particularly beneficial to neonates who often exhibit a marked inflammatory reaction and capillary leak syndrome during CPB [43]. In a study conducted by Hsia et al. with the aim of exploring the effects of aprotinin or tranexamic acid on proteolytic and cytokine profiles in infants after cardiac surgery, the expression of proinflammatory cytokines and associated matrix metaloproteinases was reduced in an aprotinin group compared with a tranexamic acid group [44]. Two currently published reports also demonstrated that aprotinin attenuates postoperative expression of pro-inflammatory factors and inflammatory gene expression whereas tranexamic acid does not [45,46]. Theses results were consistent with prior studies of cardiac surgery patients [13,14,47–49].Because of the retrospective form of the present study, we lacked data for specific biomarkers of inflammation. However, from the clinical parameters of postoperative recovery outocmes such as chest-tube drainage, duration of stay in the intensive care unit, duration of hospitalization and in-hospital mortality indicated no statistically significant differences among the three groups, suggesting that the potential antiinflammatory effect of aprotinin was clinically negligible.

One of the major side effects of aprotinin therapy in neonatal patients is renal injury. Renal injury has been extensively studied in aprotinin, because it is appropriated by the brush border of the renal tubules after filtration that has caused concerns regarding the potential for renal toxicity [50]. The possible effects of aprotinin on renal function have been both experimentally and clinically described (e.g. decreased kinin synthesis, diminished renal blood flow and glomerular filtration rate, and reversible tubular overload) [51]. In adult cardiac surgery patients, Wagener et al. observed that the use of aprotinin was associated with both an increased risk of acute renal injury and an increase of urinary neutrophil gelatinase-associated lipocalin, which is a sensitive marker for renal injury [52]. The adverse effects of aprotinin varying with the age of patients remains unsubstantiated. In a cohort study presented by Backer et al. that examined aprotinin safety in congenital heart operations, no association was observed between aprotinin use and acute renal failure, dialysis requirement, neurologic complication or mortality [53]. Another large-scale study involving 35 children’s hospitals also concluded that the use of aprotinin in congenital heart operations is safe without increased mortality or dialysis [54]. However, in a recently published study by Leyvi et al. [55], higher odds of acute renal injury were observed, compared with ε-aminocaproic acid, suggesting that the established concerns for adults with adverse kidney effects treated using aprotinin are also applicable to paediatric patients. In our study, postoperative creatinine levels after 24 hours were significantly higher than preoperative creatinine levels in all three groups. However, no evidence was found for the progression to renal failure or dialysis following administration of either aprotinin or tranexamic acid.

From the accumulated evidence, aprotinin effectively reduces blood loss and the need of blood transfusion associated with heart surgery and currently in Canada and the EU, Health Canada, and the EMA believe the benefits of aprotinin outweight its risks in isolated CABG surgery [56,57]. Wilder et al also demonstrated that aprotinin had significantly lower intraoperative transfusion requirements, surgical reexploration, renal injury and shorter surgical times in neonate heart operations [58]. These studies clearly illustrate the need for further large-scale randomized, well-designed with adequately grouped clinical trial to investigate the safety and efficacy of aprotinin in cardiac surgery.

The present report demonstrates single center data analysis that is unique and important with clinical significance. The limitations of this study include retrospective design, omitted variable bias in the database, lack of strictly defined transfusion triggers, lack of some direct measures of inflammation markers, possibility of type 2 error which is related to the power. Additionally, some factors including median cardiopulmonary bypass time and deep hypothermic circulatory arrest time may influence coagulation and fibrinolysis and thus confound the data interpretation.

## Conclusions

In conclusion, our study demonstrated that neonates who received aprotinin benefited from the blood-sparing effects without increasing the risk of acute renal injury and other adverse clinical outcomes. The data provide reasonable evidence that aprotinin and tranexamic acid are safe and efficacious as antifibrinolytic modalities in neonatal patients undergoing cardiac surgery.

## Supporting Information

S1 Data Raw Data used in this study. (XLS)
